# Lactate and cognition: a dual modulator

**DOI:** 10.3389/fnmol.2026.1742681

**Published:** 2026-02-27

**Authors:** Wen Yang, Yu Xu, Kunhua Wang

**Affiliations:** 1 Department of Clinical Psychology, The First People’s Hospital of Yunnan Province, The Affiliated Hospital of Kunming University of Science and Technology, Kunming, China; 2 Yunnan Technological Innovation Center of Drug Addiction Medicine, Yunnan University, Kunming, China; 3 The First Affiliated Hospital of Kunming Medical University, Kunming, China

**Keywords:** astrocyte–neuron lactate shuttle, cognition, epigenetic regulation, lactate, neuroinflammation, synaptic plasticity

## Abstract

Lactate, traditionally regarded as a byproduct of glycolysis, has emerged as a key metabolic substrate and signaling molecule in the brain. Through the astrocyte–neuron lactate shuttle, lactate provides an essential link between energy metabolism and neuronal function. Beyond its metabolic role, lactate influences synaptic plasticity, neuroinflammation, mitochondrial dynamics, and epigenetic regulation, thereby exerting multifaceted effects on cognitive processes. Accumulating evidence demonstrates that lactate acts as a double-edged regulator: under certain conditions, it promotes neuronal resilience and cognitive enhancement, whereas excessive accumulation or impaired transport may contribute to dysfunction. This review synthesizes current knowledge of lactate metabolism in the central nervous system, highlighting its physiological functions, bidirectional impact on cognition, and emerging role as both a biomarker and therapeutic target. A deeper understanding of lactate-mediated mechanisms may pave the way for novel strategies in the prevention and intervention of cognitive impairment. Clinically, lactate is best interpreted as a context-sensitive metabolic readout rather than a standalone disease-specific biomarker.

## 1 Introduction

Cognitive impairment refers to a decline in abilities such as thinking, learning, and memory, which severely affects quality of life. It is estimated that by 2050, the number of people living with dementia worldwide will reach 152.8 million, nearly three times the 57.4 million cases estimated in 2019, posing a major global public health challenge (GBD 2019 Dementia Forecasting Collaborators, 2022). Notably, China has the largest population of dementia patients, imposing a substantial burden on public health systems (Jia et al., 2020). Mild cognitive impairment (MCI) is recognized as an early stage of dementia, with up to 15% of patients progressing to dementia within 2 years (Alzheimers and Dementia, 2023). Preventing or delaying cognitive decline and dementia can significantly improve quality of life in late adulthood and prolong functional independence (Petersen et al., 2018). The definition of cognitive impairment encompasses a broad spectrum ranging from MCI to severe dementia, including Alzheimer’s disease (AD) and Parkinson’s disease (PD) (Wang et al., 2024). Its etiology is multifactorial, involving genetic predisposition, environmental exposures, lifestyle factors, and comorbid health conditions (Zhu et al., 2020). Pathophysiologically, the underlying mechanisms are highly complex, with inflammation, oxidative stress, mitochondrial dysfunction, and blood–brain barrier disruption all implicated in disease progression (Kaur and Sharma, 2022).

Lactate, the end product of glycolysis, has long been a controversial subject in biology and exercise physiology. For more than 200 years, lactate was regarded merely as a “metabolic waste” in muscle, associated with fatigue and soreness (Ferguson et al., 2018). This view shifted in the mid-1980s when George Brooks proposed the “lactate shuttle” theory (Brooks, 1986). At its core, this theory posits that lactate functions as an energy intermediate, produced in tissues with high glycolytic activity and consumed by tissues with high oxidative capacity (Brooks et al., 2022; Brooks, 2018). Current evidence indicates that lactate serves as a metabolic bridge between glycolysis and mitochondrial respiration, acting as both a downstream product of glycolysis and a substrate for oxidative metabolism (Brooks, 2018). Importantly, according to the lactate shuttle hypothesis, this process occurs under fully aerobic conditions and can transcend cellular compartments, functioning across cells, tissues, and organs (Brooks, 2002, 2009). In the central nervous system (CNS), lactate has been recognized as both a crucial energy substrate and a signaling molecule (Magistretti and Allaman, 2018; Perry et al., 2016). It is derived from both glycolysis (Rogatzki et al., 2015) and gut microbiota (Bruning et al., 2019), and is markedly released during exercise (Goodwin et al., 2007). In the CNS, lactate functions as a rapid excitatory signal (Tang et al., 2014; Yang et al., 2014), exerting either neuroprotective (Berthet et al., 2009) or neurotoxic (Lama et al., 2014) effects depending on its concentration and duration of exposure. The astrocyte–neuron lactate shuttle (ANLS) hypothesis proposes that lactate is exported by astrocytes and subsequently taken up and oxidized by neurons, particularly in the context of glutamatergic signaling (Pellerin et al., 1998). Consistent with this concept (Barros, 2013; Bélanger et al., 2011), neurons express the molecular machinery required for glucose uptake and intracellular lactate utilization (Hashimoto et al., 2008). At the brain level, glucose–lactate interactions are critical for both physiological and pathological states. Lactate metabolism contributes to normal brain functions, including energy supply (Theparambil et al., 2024), maintenance of metabolism under hypoglycemia (Herzog et al., 2013), neurometabolic coupling and signal transduction (Li et al., 2025; Liu et al., 2017), and executive function (Hashimoto et al., 2018).

In the context of cognitive disorders, dysregulation of lactate metabolism has been linked to multiple conditions, including Alzheimer’s disease, Parkinson’s disease, traumatic brain injury, stroke, psychiatric disorders, and substance use disorders. This association underscores lactate metabolism as a fundamental biological basis for maintaining cognitive function and highlights its potential as an early diagnostic biomarker, prognostic indicator, and therapeutic target. With the advancement of technologies such as magnetic resonance spectroscopy and protein lactylation assays, dynamic monitoring of brain lactate metabolism and its molecular mechanisms has become increasingly feasible, establishing lactate as a growing focus in neuroscience and translational medicine. This review aims to provide a comprehensive overview of the relationship between lactate metabolism and cognition, spanning from fundamental mechanisms to clinical implications. We first introduce the physiological processes and regulatory mechanisms of lactate metabolism in the brain, followed by an exploration of its roles in energy supply, signaling pathways, synaptic plasticity, neurotransmission, and epigenetic regulation. On this basis, we integrate findings from both animal models and clinical studies across diverse conditions and physiological states, critically evaluate the current evidence, and propose future directions for research and clinical translation. Our goal is to provide insights that may advance mechanistic understanding and facilitate the development of novel strategies for the prevention and treatment of cognitive impairment.

Because lactate alterations are observed across many neurological and systemic conditions, lactate should not be framed as a universal “disease-specific” marker. Rather, lactate is best conceptualized as a context-sensitive readout of the brain’s metabolic state that becomes clinically informative only when interpreted within (i) disease stage (e.g., compensation vs. overload vs. exhaustion), (ii) brain-region specificity (network- and cell-type vulnerability), and (iii) sampling compartment and modality (brain tissue vs. cerebrospinal fluid (CSF) vs. plasma; Magnetic Resonance Spectroscopy (MRS) vs. metabolomics). In this framework, the same direction of lactate change may carry distinct biological meaning across disorders, while disease-relevant signatures emerge from patterned combinations–such as lactate together with monocarboxylate transporter (MCT)/lactate dehydrogenase (LDH) expression, redox/mitochondrial indices, inflammatory markers, and lactylation readouts–and from longitudinal or challenge-based dynamics (exercise, hypoxia, glycemic stress).

To increase transparency, we briefly describe our literature identification process. We searched PubMed, Web of Science, and Scopus for English-language studies published between 2015 and 2025 using keyword related to lactate metabolism, MCTs/ANLS, and cognition. We prioritized original cell/animal/clinical studies relevant to cognitive outcomes and excluded papers not addressing brain lactate biology or cognition.

## 2 Physiology and regulation of brain lactate metabolism

### 2.1 Brain energy metabolism and the astrocyte–neuron lactate shuttle

Although the brain accounts for only ∼2% of total body weight, it consumes 20%–25% of the body’s energy to sustain its function. More than 10% of cardiac output is directed to cerebral blood flow, reflecting the brain’s high demand for glucose and oxygen (Magistretti and Allaman, 2015). Glucose is the principal energy substrate for mammalian cells. In the brain, glucose is almost completely oxidized to CO2 and H2O through sequential processes including glycolysis, the tricarboxylic acid (TCA) cycle, and oxidative phosphorylation. In astrocytes, glucose metabolism predominantly occurs through aerobic glycolysis, producing lactate, which is then taken up by neurons and converted to pyruvate for oxidation via the TCA cycle and the electron transport chain (Bélanger et al., 2011; Calì et al., 2019; Magistretti et al., 1999). Lactate thus serves as a key mediator of metabolic cooperation between astrocytes and neurons. The astrocyte–neuron lactate shuttle (ANLS) model posits that lactate produced by astrocytes is an essential energy substrate for neurons (Wu et al., 2023). During glucose deprivation, astrocyte-derived lactate acts as a neuroprotective metabolite, and exogenous lactate administration can restore neuronal activity (Sun et al., 2020). In addition to being an energy source, lactate functions as a signaling molecule or receptor agonist, modulating neuronal excitability, synaptic plasticity, and cognitive processes (Magistretti and Allaman, 2018). Evidence suggests that astrocytic lactate release and subsequent neuronal uptake are indispensable for learning, memory consolidation, and long-term potentiation (LTP) (Vezzoli et al., 2020). Suzuki et al. (2011) demonstrated that glycogenolysis and astrocytic lactate release are required for long-term memory formation. Thus, lactate contributes to learning and memory through redox- and energy-dependent mechanisms, highlighting its potential as a therapeutic target.

### 2.2 Transmembrane transport of lactate

The transport of lactate across cell membranes is mediated by monocarboxylate transporters (MCTs), a family of 14 transmembrane proteins (MCT1–14; SLC16A1–A14) that facilitate the movement of lactate, pyruvate, and β-hydroxybutyrate (Bergersen, 2015). In the CNS, three isoforms–MCT1, MCT2, and MCT4–show distinct regional and cellular distributions (Eid et al., 2018). Lactate transfer through the ANLS and MCTs exhibits cellular specificity: astrocytes express low levels of MCT1 and MCT4, while neurons express the high-affinity transporter MCT2, reflecting a specialized division of labor (Pierre and Pellerin, 2005). Neuronal MCT2, primarily located at postsynaptic membranes, facilitates the uptake of lactate, pyruvate, and ketone bodies as energy substrates (Pierre et al., 2002). Disruption of astrocytic lactate production or downregulation of astrocytic MCT1 and MCT4 expression in the hippocampus impairs long-term memory formation. Notably, this impairment can be reversed by exogenous L-lactate administration, underscoring the critical role of astrocyte–neuron metabolic cooperation in maintaining cerebral energy demands, redox homeostasis, and neurotransmitter receptor activity (Bonvento and Bolaños, 2021).

### 2.3 Metabolic pathways of lactate in the brain

Recent studies have established lactate as a key player in memory formation and neuroprotection (Proia et al., 2016). Lactate in the brain originates both from central glycolytic activity and peripheral sources transported across the blood–brain barrier. Within the CNS, lactate is primarily generated in astrocytes through glycolysis and glycogenolysis, and it plays a central role in astrocyte–neuron metabolic coupling (Dias et al., 2023). Astrocytes are crucial for brain metabolism: via glucose transporter 1 (GLUT1)-mediated glucose uptake, they metabolize glucose through glycolytic enzymes such as hexokinase (HK), phosphofructokinase (PFK-1) and its regulator PFKFB3, as well as pyruvate kinase M2 (PKM2), producing pyruvate (Takahashi, 2021). Pyruvate is then converted into lactate by lactate dehydrogenase A (LDHA), regenerating NAD^+^ to maintain energy balance (Denker and Dringen, 2024). Astrocytic glycogen reserves can be rapidly mobilized under norepinephrine stimulation, with glycogen phosphorylase (PYGB) releasing glucose-1-phosphate that enters glycolysis to generate abundant lactate (Coggan et al., 2018). Lactate is subsequently exported from astrocytes through MCT1 and MCT4, supplying energy substrates to nearby active neurons (Descalzi et al., 2019).

Neurons utilize high-affinity MCT2 transporters to import astrocyte-derived lactate. Once inside neurons, lactate is oxidized to pyruvate by lactate dehydrogenase B (LDHB), generating NADH for downstream oxidative metabolism (Descalzi et al., 2019; Lee et al., 2025). Pyruvate is then converted into acetyl-CoA by the pyruvate dehydrogenase (PDH) complex and enters the TCA cycle, driving oxidative phosphorylation and ATP production to support action potentials, neurotransmitter release, and synaptic plasticity (Tiwari et al., 2024). Increased synaptic activity leads to massive glutamate release, which is taken up by astrocytes via excitatory amino acid transporter 1 (EAAT1/GLAST) and excitatory amino acid transporter 2 (EAAT2/GLT-1). This process elevates Na^+^/K^+^-ATPase activity and energy consumption (Gegelashvili et al., 2007; Robinson and Jackson, 2016), thereby stimulating glycolysis and lactate production. Lactate exported via astrocytic MCT1/4 is rapidly taken up and oxidized by neurons through MCT2, not only fulfilling energy requirements but also activating signaling cascades that facilitate LTP and memory consolidation (Suzuki et al., 2011; Yang et al., 2014).

**FIGURE 1:**
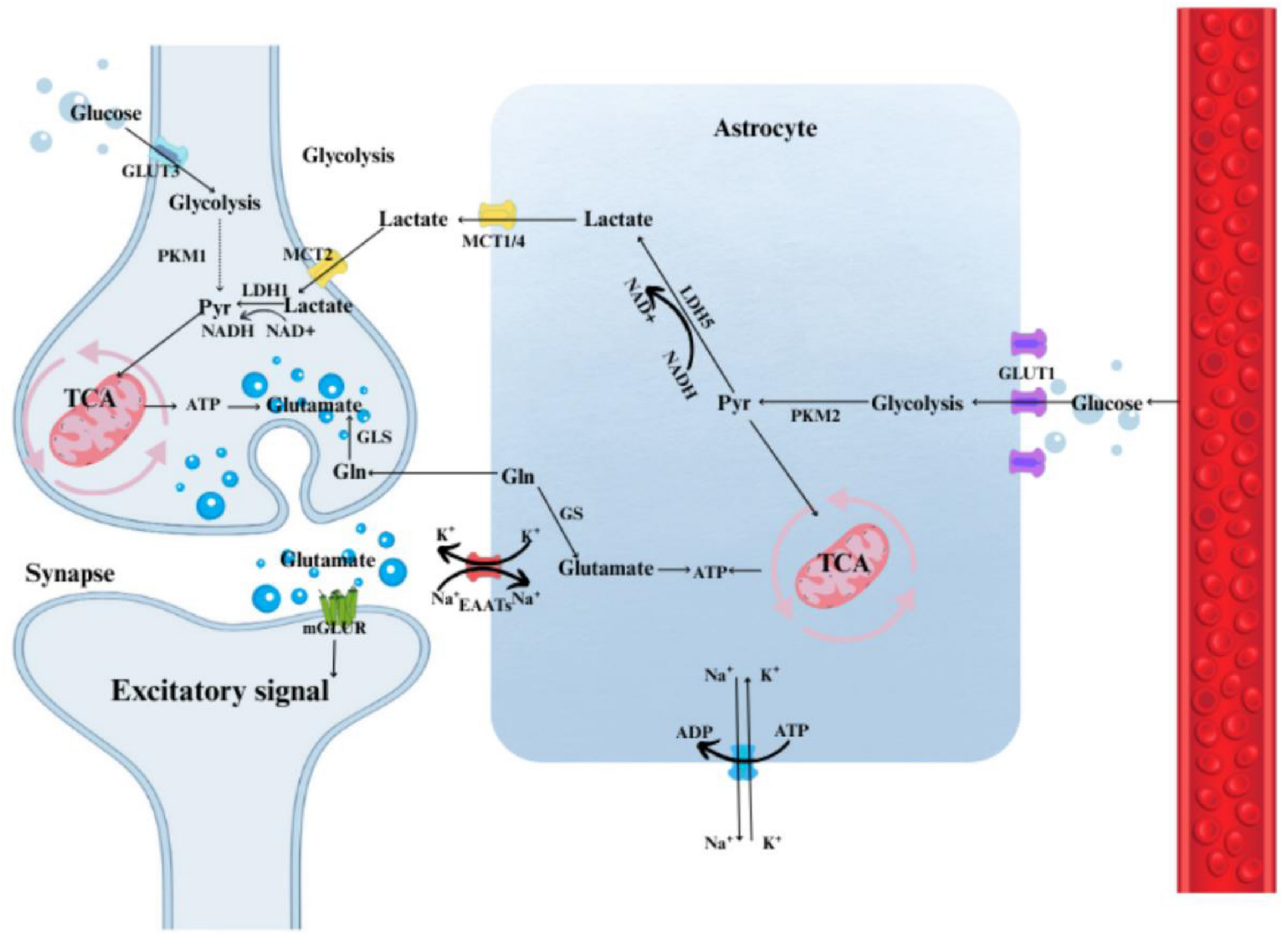
Lactate metabolism in the brain and the astrocyte–neuron lactate shuttle. Schematic illustration of lactate metabolism in the brain and the astrocyte–neuron lactate shuttle (ANLS). Glucose enters astrocytes via GLUT1, where it undergoes glycolysis and glycogenolysis to generate lactate. Lactate is exported through MCT1 and MCT4 and subsequently taken up by neurons via MCT2, where it is converted into pyruvate and enters the TCA cycle to support oxidative phosphorylation. This metabolic coupling provides energy for synaptic activity and contributes to memory formation and cognitive function.

Multiple key enzymes, transporters, and receptors are involved in lactate regulation, including glycolytic enzymes HK1/2 (Nowak et al., 2018), PFK-1, PFKFB3 (Imbert-Fernandez et al., 2024; Xiao et al., 2023), and PKM2/PKM1 (Fukushi et al., 2022), lactate dehydrogenase isoforms LDHA (Tian et al., 2023) and LDHB (Park et al., 2022), PDH and its regulators PDK/PDP (Zhou et al., 2025), and glycogen metabolism enzymes GYS1 (Nitschke et al., 2025) and PYGB (Yang C. et al., 2024). Lactate receptor hydroxycarboxylic acid receptor 1 (HCAR1/GPR81), a Gi/o-coupled receptor, suppresses cAMP signaling, contributing to neurovascular coupling and protection against excitotoxicity (Colucci et al., 2023). More recently, lysine lactylation of proteins has emerged as a novel epigenetic mechanism linking lactate to inflammation regulation (Yu F. et al., 2025) and synaptic plasticity (Wu et al., 2024). Collectively, lactate is not only an intermediate metabolite in energy metabolism but also an active regulator in diverse pathophysiological processes within the brain (Figure 1).

## 3 Mechanisms of lactate in cognitive function

Current evidence indicates that lactate exerts a “double-edged” effect on cognition, with outcomes that can be beneficial or detrimental depending on disease context. This heterogeneity is shaped by lactate concentration and temporal dynamics, regional and compartmental specificity, neuron–glia metabolic coupling, and the organism’s adaptive capacity to metabolic stress. In addition to serving as an energy substrate, lactate also acts as a signaling molecule and an epigenetic regulator, thereby influencing synaptic plasticity, neuroinflammation, mitochondrial function, and gene expression.

Mechanistic boundary conditions for lactate’s beneficial versus detrimental effects. Lactate-related cognitive effects can be reconciled by four interacting determinants: (i) concentration and dynamics (moderate/transient vs. sustained accumulation vs. depletion), (ii) duration of exposure (acute vs. chronic), (iii) cellular source and site of action (astrocyte-to-neuron shuttle vs. maladaptive glycolytic overdrive; tissue vs. CSF vs. plasma), and (iv) disease stage and metabolic capacity (preserved vs. impaired transport–utilization coupling). Because absolute lactate values vary substantially across assays, compartments, and protocols, we do not propose a universal numeric threshold. Instead, we interpret lactate based on directionality, duration, and coupling integrity, and summarize condition-specific patterns in Tables 1, 2. In general, lactate is most likely beneficial when it restores astrocyte–neuron metabolic coupling (ANLS) and supports neuronal oxidative capacity, but becomes detrimental when production exceeds utilization/clearance or when chronic exposure triggers maladaptive signaling and pathogenic protein modifications.

**TABLE 1:** Comparative lactate dynamics across diseases, stages, and brain regions relevant to cognition.

Disease	Stage	Key brain region(s)	Lactate change	Net interpretation for cognition	Key accompanying features
AD	Early/MCI	Hippocampus, frontal cortex	↑ (often)	Initially compensatory; may become maladaptive if sustained	Lactate rises prior to Aβ deposition with enhanced astrocytic glycolysis; accumulation can drive A1 astrocyte activation via AKT–mTOR–HIF-1α; rapamycin mitigates
AD	Early (subset reports)	Astrocytes, region-dependent	↓ (reported)	Early glycolytic suppression may impair support	Oxidative stress and glycolytic suppression in AD astrocytes may reduce lactate release
AD	Late/dementia	Cognition-related regions	↓	Metabolic exhaustion; impaired plasticity/memory	Lactate decline with MCT2 downregulation; suppressed astrocytic glycolysis; LDHA↓; IDO1–KYN–AhR axis; ubiquitination-mediated LDHA degradation; lactate depletion reduces APP-K612 lactylation and increases Aβ
AD	Pathogenic lactylation arm	Molecular	↑	Harmful signaling	Excess lactate can promote tau pathology via p300-mediated tau K331 lactylation; MAO-B/oxidative stress
DACD (T1D)	Very early (as early as week 3)	Hippocampus, hypothalamus, striatum, cortex	↑	Potential early biomarker; may transiently support function	Elevation precedes overt cognitive impairment
DACD (T1D)	Progression	Hippocampus, cortex	↑↑ (accumulation)	More likely harmful when clearance/utilization fails	LDHB activity↓ + MCT2↓ suggests overproduction + impaired clearance; FGF21 improves cognition by upregulating MCT2 and LDHB via PI3K/Akt/mTOR
DACD (stress condition)	Recurrent hypoglycemia	Brain	↓	Biphasic pattern; stress-stage dependent	Lactate declines during recurrent hypoglycemia, contrasting typical diabetes elevation
TBI	Early/acute (some reports)	Brain; cortex, hippocampus (intervention)	↓	Lactate supplementation beneficial (metabolic support)	Hypertonic sodium lactate reverses depletion and improves energy and function; lactate preconditioning via GPR81 improves plasticity/cognition
TBI	Acute, variable	Brain	↑ (some reports)	Possibly compensatory; context-dependent	LLL (low-level light) + lactate/pyruvate improves mitochondrial function, protects hippocampus, restores cognition
POCD	Postoperative multiple time points	Hippocampus	↓	Energy supply deficit → cognitive decline	Surgery-induced inflammation reduces hippocampal lactate; H2S restores Warburg effect and synaptic plasticity
POCD (aged)	Certain postoperative stages	Brain/hippocampus	↑	Can be protective if moderate; harmful if accumulation-driven	Lactate supplementation improves cognition via SIRT1 (blocked by EX-527); fructose pathway activation increases lactate synthesis and worsens cognition; inhibition lowers lactate and improves
Exercise (acute)	Post-exercise	Systemic, hippocampus	↑	Often beneficial; depends on intensity/clearance/age	Lactate elevation associated with executive function changes; lactate activates SIRT1/PGC1α/BDNF and may reshape inflammatory phenotypes
Aging	Young vs. aged	Hippocampus	Bidirectional	Young: lactate supports LTP; aged: accumulation may be detrimental	Young: lactate essential for LTP; aged: inhibiting lactate production can improve LTP; aerobic glycolysis increases with aging
Stroke/ischemia	Acute	Astrocyte PKM2 dependent	Supply disruption	Lactate supplementation beneficial	Astrocytic PKM2 loss disrupts lactate energy supply; exogenous lactate reverses neuronal death/cognitive deficits
aSAH	Metabolic crisis	Brain tissue (CMD)	↑ (often high)	High lactate linked to poor outcomes	High lactate correlates with cerebral microdialysis (CMD) total-tau; linked to hypoxia/axonal injury/poor cognition
Schizophrenia	Across stages	Anterior cingulate cortex	↑	Generally adverse association	Elevated lactate negatively correlates with cognitive/functional scores; stage differences noted
ASD/developmental disorders	Developmental	Plasma, systemic	↑	Higher lactate linked to poorer cognition/adaptive ability	Plasma lactate elevated; negative correlation with cognitive/adaptive abilities

↑/↓ indicates the direction reported in the cited studies; interpretation depends on stage, region, and transport–utilization capacity. AD, Alzheimer’s disease; DACD, diabetes-associated cognitive dysfunction; POCD, postoperative cognitive dysfunction; TBI, traumatic brain injury; aSAH, aneurysmal subarachnoid hemorrhage.

**TABLE 2:** Beneficial versus harmful lactate signaling across disease stages: a practical decision matrix.

Feature	Beneficial lactate signaling (typically early/acute or well-coupled)	Harmful lactate signaling (typically overload/mismatch)	Exhaustion phenotype (late/chronic failure)
Lactate pattern	Moderate/transient ↑	Sustained ↑/accumulation	↓/depletion
ANLS/transport–utilization coupling	Coupled shuttle; neuronal uptake/oxidation preserved (e.g., adequate MCT2/LDHB)	Mismatch: production exceeds utilization (e.g., MCT2↓, LDHB↓)	Supply failure: LDHA↓/MCT2↓, suppressed astrocytic glycolysis
Dominant role	Metabolic fuel supporting LTP/plasticity and cognition	Metabolic stress marker + maladaptive signaling (inflammation/oxidative stress/protein lactylation)	Energy insufficiency → synaptic failure, LTP impairment, cognitive decline
Glial/inflammation context	Can promote reparative shifts (exercise-related microglial phenotype changes)	A1 astrocyte activation and feed-forward glycolysis amplification (e.g., AKT–mTOR–HIF-1α in AD)	Chronic metabolic collapse with impaired glial support
Lactylation-related examples	Protective arm: APP-K612 lactylation promotes lysosomal degradation and reduces Aβ; lactate can enhance	Pathogenic arm: p300-mediated tau K331 lactylation increases phosphorylation/aggregation; MAO-B/oxidative stress	Reduced lactate may also reduce beneficial lactylation programs
Representative mappings	Early AD/MCI; acute TBI with lactate depletion rescued by supplementation; exercise-induced lactate	Subarachnoid hemorrhage (aSAH) metabolic crisis (high lactate + tau/poor outcome); POCD fructose-pathway lactate accumulation; AD overload states	Late AD dementia (lactate↓ + MCT2↓ + LDHA↓); POCD hippocampal lactate↓
Practical implication	Support lactate availability/uptake when depletion exists; preserve coupling	Reduce drivers of accumulation; restore utilization capacity	Restore synthesis/transport (LDHA/MCT2 axis) and protect synapses

↑, increase in lactate; ↓, decrease in lactate; →, leads to/results in (causal direction).

**FIGURE 2:**
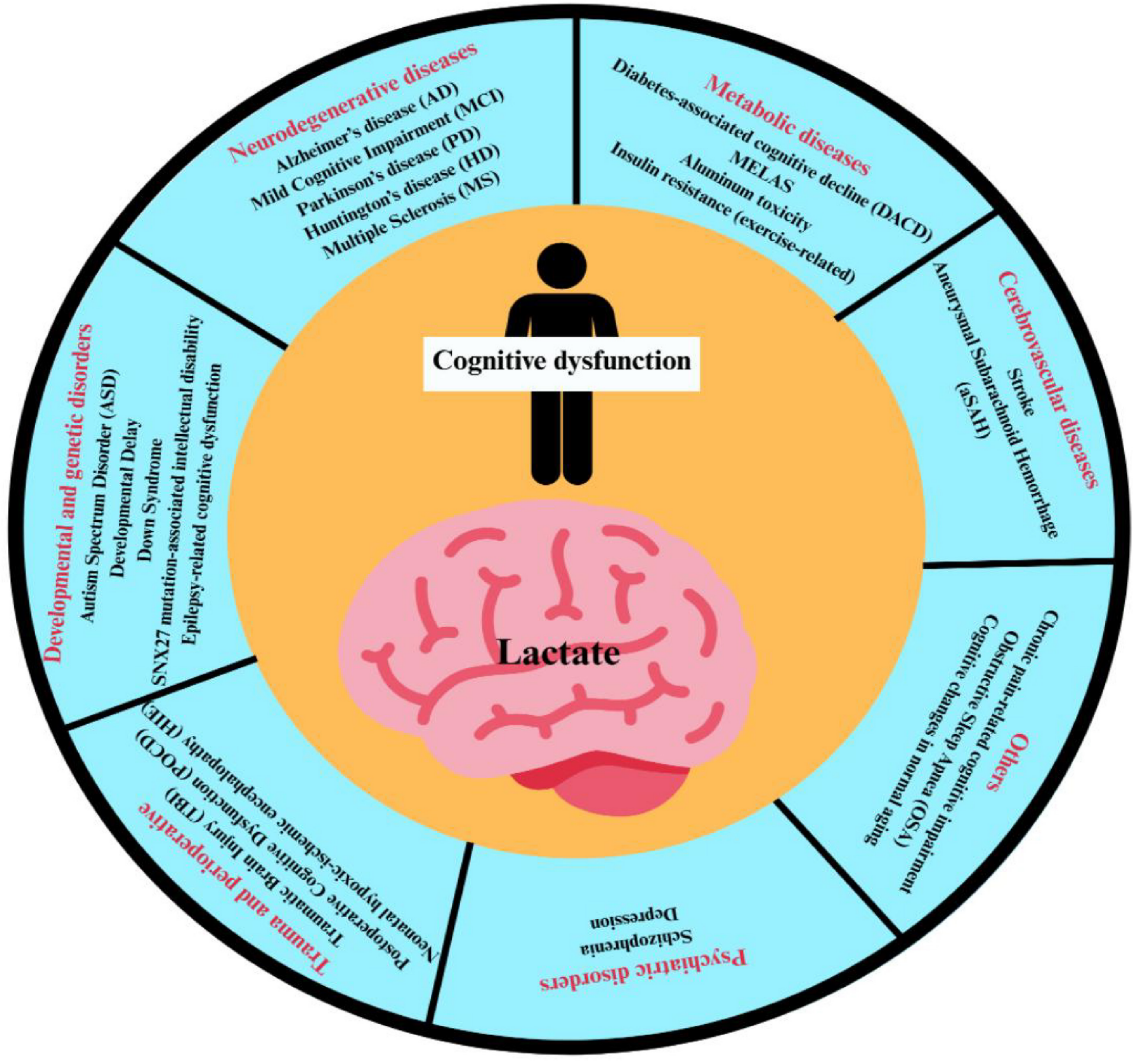
Lactate-associated cognitive disorders.

Consistent with this framework, the recurrent cognitive domains and their mappings across conditions are summarized in Figure 2, while condition-specific lactate trajectories are synthesized in Tables 1, 2.

### 3.1 Alzheimer’s disease (AD)

Alzheimer’s disease (AD) is the most common neurodegenerative disorder, characterized by β-amyloid (Aβ) deposition (Magalingam et al., 2018), tau hyperphosphorylation (Muralidar et al., 2020), synaptic dysfunction (Knopman et al., 2021), and neuroinflammation (Kloske and Wilcock, 2020). Lactate is no longer viewed solely as a metabolic byproduct but as a regulator of brain energy metabolism, signaling, and epigenetic modulation. In AD, these functions are closely tied to astrocyte–neuron metabolic coupling and may contribute to energy deficits and cognitive decline. Evidence suggests a stage-dependent relationship between lactate and cognitive impairment in AD. In the early stage and mild cognitive impairment (MCI), lactate levels are often elevated. Preclinical evidence from cellular and animal models indicates that in multiple AD mouse models, lactate concentrations–especially in the hippocampus and frontal cortex–rise prior to Aβ deposition, accompanied by enhanced astrocytic glycolysis (Harris et al., 2016; Santos et al., 2022; Yang X. et al., 2024; Zhu et al., 2025). In microglia exposed to AD plasma, glycolytic enzymes such as glyceraldehyde-3-phosphate dehydrogenase and pyruvate kinase are upregulated, increasing lactate production and apoptosis, thereby impairing cellular energy metabolism (Jayasena et al., 2015). Mechanistically, enhanced astrocytic glycolysis and lactate export may initially act as a compensatory response to energy stress. However, sustained lactate accumulation may promote A1 astrocytic activation via AKT–mTOR–HIF-1α signaling, further amplifying glycolysis and lactate production. This feed-forward loop has been linked to impaired LTP and increased Aβ aggregation and can be attenuated by rapamycin (Harris et al., 2016; Yang X. et al., 2024). Nevertheless, some studies report early lactate reductions, suggesting that glycolytic suppression and oxidative stress in AD astrocytes may lead to decreased lactate release and cognitive impairment (Oksanen et al., 2017; Tarczyluk et al., 2015). In the late stage and dementia phase, lactate decline and downregulation of MCT2 indicate impaired energy metabolism, correlating with advanced pathology and memory deficits (Lu et al., 2015). In late-stage AD brains, reduced lactate is associated with suppressed astrocytic glycolysis, LDHA downregulation, and activation of the IDO1–KYN–AhR axis, all of which exacerbate neuronal energy deficits and LTP impairment (Minhas et al., 2024; Sun et al., 2020). Mechanisms such as VGLL4 downregulation and increased ubiquitination-mediated degradation of LDHA may further reduce lactate synthesis (Tian et al., 2023). Lactate transport dysfunction is another pathogenic mechanism: overexpression of MCT4 in astrocytes increases lactate export but disrupts neuronal energy homeostasis, impairing function and survival (Hong et al., 2020). At the molecular level, under certain conditions, excess lactate can promote tau pathology through p300-mediated tau lactylation at K331, enhancing tau phosphorylation and aggregation (Zhang X. et al., 2025), as well as upregulating MAO-B expression and oxidative stress (Lee et al., 2018). Clinically, observational studies further suggest that CSF lactate is significantly elevated in the MCI stage (Zebhauser et al., 2022), and large-scale studies indicate that CSF lactate levels fluctuate across the AD continuum–elevated in MCI but often declining as dementia progresses–reflecting a shift from metabolic overactivation to metabolic exhaustion; notably, lactate inversely correlates with tau pathology, suggesting that its dynamics may mirror neuronal metabolic impairment in AD (Bonomi et al., 2021; Liguori et al., 2015). Interventional and translational studies provide preliminary causal support. For example, rapamycin mitigates lactate-linked A1 astrocytic activation and AKT–mTOR–HIF-1α signaling (Harris et al., 2016; Yang X. et al., 2024), separately, lactate depletion can reduce APP-K612 lactylation, whereas exogenous L-lactate enhances APP lactylation, consistent with a potential role for lactylation in modulating Aβ generation (Zhang X. et al., 2025); In parallel, lactate serves as both an energy substrate and a neuroprotective factor; under metabolic stress, moderate supplementation can improve synaptic plasticity and cognition (Wang et al., 2025). Exercise or exogenous lactate elevates brain lactate, enhances histone H3 lactylation (H3Kla), and induces a microglial phenotype shift from pro-inflammatory to reparative, thereby reducing neuroinflammation and improving cognition (Han et al., 2023). Conversely, inhibition of MCT4 reduces neuronal apoptosis and inflammation, ultimately preserving LTP and memory (Hong et al., 2020). A stage-resolved synthesis of AD-related lactate findings (including early/MCI versus late dementia patterns) is summarized in Table 1 and interpreted using the decision matrix in Table 2.

In summary, lactate levels in AD exhibit stage-dependent changes: elevated in early stages but reduced in late stages due to impaired synthesis and transport. Lactate participates in multiple pathological processes–including energy metabolism, inflammatory states, synaptic plasticity, tau pathology, and APP processing–with astrocytic metabolic reprogramming as a central driver. Lactate may act both as a pathological mediator and as a therapeutic target, reflecting its double-edged properties. Dynamic lactate monitoring thus holds promise as an early diagnostic and stratification biomarker in AD, particularly when combined with stage definitions and complementary AD-relevant markers (e.g., Aβ/tau) and region-specific readouts.

For rapid cross-condition comparison of lactate directionality, key brain regions, and cognitive interpretation across the disorders covered, please refer to Table 1.

### 3.2 Diabetes-associated cognitive dysfunction (DACD)

Diabetes-associated cognitive dysfunction (DACD) has increasingly been recognized as a critical complication of diabetes, exerting profound effects on patients’ quality of life. Metabolic disturbances in diabetes may disrupt regional brain energy balance and synaptic plasticity, thereby contributing to the onset and progression of cognitive impairment. Across T1D models, multiple studies report elevated brain lactate, particularly in cognition-related regions (e.g., hippocampus, hypothalamus, striatum, and cortex) (Ando et al., 2022; Dong et al., 2019; Zhang et al., 2020; Zhao et al., 2018, 2022; Zheng et al., 2017a). These changes are commonly linked to disrupted neuron–glia metabolic coupling and neurotransmitter imbalance, which together may contribute to cognitive deficits. Remarkably, lactate elevation occurs as early as the third week, before overt cognitive impairment develops, suggesting its potential role as an early metabolic biomarker (Ando et al., 2022). As the disease progresses, lactate accumulation intensifies, accompanied by reduced LDHB activity and decreased MCT2 expression. This indicates a dual pathology of excessive lactate production and impaired clearance. Notably, fibroblast growth factor 21 (FGF21) ameliorates learning and memory deficits in DACD mice by enhancing neuronal lactate uptake (via MCT2 upregulation) and utilization (via LDHB upregulation) (Zhao et al., 2022). Mechanistically, this effect involves PI3K/Akt/mTOR-dependent translation of MCT2, which promotes pyruvate generation and ATP/NADH production and helps restore hippocampal energy metabolism and synaptic plasticity. Other studies, however, suggest that early in diabetes, astrocytic metabolism is initially upregulated, leading to increased lactate that temporarily supports neuronal function; yet with disease progression, astrocytic support diminishes, reflecting metabolic failure in the diabetic brain (Wang et al., 2015). Interestingly, during recurrent hypoglycemia, brain lactate levels decline rather than rise, indicating biphasic changes in lactate metabolism across different pathological stages and stress conditions (Wu et al., 2025). In type 2 diabetes (T2D), lactate changes appear more heterogeneous across models; nevertheless, many studies report elevated lactate in brain tissue, particularly in the hippocampus (Hackett et al., 2019; Shima et al., 2017; Zheng et al., 2016, 2017b,c). Stress-related increases in amygdalar lactate further disrupt energy homeostasis, providing a metabolic basis for cognitive dysfunction under diabetic stress (Xu et al., 2019). Lactate accumulation is accompanied by enhanced glycolysis, increased activity of lactate-alanine shuttling, and disrupted neuron–astrocyte metabolic communication. As a result, lactate becomes inefficiently utilized for energy production and neurotransmitter synthesis, leading to energy network reprogramming and cognitive decline (Zheng et al., 2017b). At the epigenetic level, histone lactylation (e.g., H4K12la) is upregulated under diabetic conditions, activating the FOXO1/PGC-1α pathway, which exacerbates mitochondrial oxidative stress and neuronal apoptosis, providing molecular evidence of lactate’s pathogenic role (Yang et al., 2025). Importantly, even at the prediabetic stage (e.g., 6 months of high-fat diet), brain metabolic changes are evident, suggesting that lactate dysregulation may precede clinical cognitive decline (Choi et al., 2019). Clinically, observational studies indicate that DACD manifests as impairments in attention, memory, executive function, visuospatial skills, and language abilities (Yu X. et al., 2025). Interventional and translational studies provide preliminary causal support showing that lactate modulation may have therapeutic potential: intracerebroventricular lactate injection improves cognition in diabetic mice (Kobayashi et al., 2019; Wu et al., 2025), and lactate supplementation alleviates recurrent hypoglycemia-induced brain dysfunction by restoring ANLS, reducing oxidative stress, and supporting mitochondrial function and synaptic plasticity (Wu et al., 2025). Exercise exerts similar benefits by increasing lactate production, enhancing lactate-dependent mitophagy, and activating the lactate–SIRT1–FOXO3–PINK1/Parkin axis, thereby improving T2D-related cognitive dysfunction (Khosravi et al., 2024). Exercise may also promote lactate transport by upregulating MCT2 (Shima et al., 2017), enhances BDNF expression (Jesmin et al., 2022), restores hippocampal lactate metabolism, and attenuates diabetes-associated cognitive decline (Rooijackers et al., 2017).

In summary, diabetes–particularly at stages of cognitive dysfunction–is consistently associated with lactate metabolic abnormalities, including lactate accumulation, pathway dysregulation, and shuttle impairment. Lactate is not merely a passive byproduct but also an active pathogenic factor that contributes to cognitive decline by disrupting mitochondrial function, synaptic plasticity, and epigenetic regulation.

### 3.3 Exercise-related studies

Exercise is associated with improvements in multiple cognitive domains, most consistently executive function, attention, and memory. However, evidence linking peripheral lactate dynamics to cognitive outcomes is heterogeneous across exercise intensity, age, and lactate clearance capacity. Preclinical studies indicate that exercise increases hippocampal lactate, accompanied by activation of the SIRT1/PGC1α/BDNF pathway and improved learning and memory (El Hayek et al., 2019). Exogenous L-lactate also promotes adult neurogenesis; however, without concurrent training, gains in spatial cognition appear limited (Lev-Vachnish et al., 2019). Clinically, observational studies in humans suggest that exercise–especially high-intensity interval training (HIIT)–leads to significant increases in peripheral blood lactate levels. Importantly, these elevations have shown bidirectional effects on cognition. Several studies report that elevated lactate is positively associated with improved cognitive performance, particularly in executive function, attention, and semantic fluency (Ballester-Ferrer et al., 2022; Dora et al., 2021; Herold et al., 2022; Kujach et al., 2020; Li et al., 2024; Oliva et al., 2023; Tomoo et al., 2021). For example, post-HIIT lactate elevation has been positively correlated with improvements in executive function (Hashimoto et al., 2018; Oliva et al., 2023). Blood lactate has been identified as a key mediator in enhancing attention (Herold et al., 2022), and lactate release following intense exercise is associated with reduced cognitive reaction times (Li et al., 2024). Conversely, several studies link higher lactate during strenuous or high-intensity exercise to transient decrements in working memory, attention, or overall cognitive performance (Coco et al., 2020a; Perciavalle et al., 2015). Young adults appear more susceptible to lactate-related cognitive effects than older individuals (Coco et al., 2020b), whereas several studies report no cognitive improvement despite elevated lactate (Oberste et al., 2016; Sudo et al., 2017). Notably, lactate clearance rate–rather than peak lactate–appears to better predict cognitive improvement (Zimmer et al., 2016). Professional athletes may exhibit adaptive regulatory mechanisms that increase tolerance to elevated lactate or even confer neuroprotection (Coco et al., 2019). Interventional and translational studies provide preliminary causal support: lactate supplementation offsets cognitive decline induced by repeated HIIT sessions (Cho et al., 2020), and sodium bicarbonate promotes lactate efflux and improves cognitive performance (Chycki et al., 2021).

Taken together, lactate–a key metabolite elevated following exercise–plays a multifaceted role in cognitive regulation. On one hand, exercise-induced lactate elevation may enhance brain energy metabolism and synaptic plasticity, thereby improving attention and executive functions. On the other hand, excessive lactate accumulation or inefficient clearance may be linked to transient impairments in working memory and other cognitive domains. Thus, the cognitive effects of lactate are bidirectional, shaped by factors such as fitness level, exercise intensity, age, lactate metabolism, and neurophysiological adaptability.

### 3.4 Trauma, surgery, and anesthesia

Lactate dynamics following traumatic brain injury (TBI) exhibit complex temporal patterns. Preclinical evidence from cellular and animal models indicates that early after TBI, brain lactate may decrease. Hypertonic sodium lactate supplementation can reverse lactate depletion, improve energy status, and restore neurological function, supporting a potential metabolic and neuroprotective role (Millet et al., 2018). Lactate preconditioning via GPR81 activation upregulates plasticity-related proteins in the cortex and hippocampus, improves cognition and synaptic plasticity, and reduces neuronal injury in TBI models (Zhai et al., 2020). Alternatively, some studies report elevated lactate after TBI, potentially reflecting enhanced astrocytic glycolysis and/or compensatory mitochondrial metabolism. Notably, combined low-level light therapy (LLLT) with lactate or pyruvate enhances mitochondrial function, protects hippocampal tissue, and restores cognition (Dong et al., 2015). Similarly, repeated neonatal sevoflurane exposure induces cognitive impairment in adult male mice, along with reduced hippocampal neurogenesis and impaired synaptic plasticity; lactate intervention reverses these deficits, but only in males (Qiu et al., 2023). In mouse models, surgery triggers systemic inflammation leading to reduced hippocampal lactate at multiple postoperative time points, impairing energy supply and inducing cognitive decline (Femenía et al., 2018). Lactate depletion disrupts synaptic plasticity and memory performance, whereas hydrogen sulfide alleviates postoperative cognitive dysfunction (POCD) by promoting the Warburg effect and restoring hippocampal synaptic plasticity (Chen et al., 2020). Other studies report elevated lactate at certain postoperative stages, particularly in aged mice. In these models, lactate supplementation mitigates anesthesia- and surgery-induced cognitive impairment, accompanied by reduced oxidative stress and neuroinflammation and restored synaptic protein expression. These effects appear SIRT1-dependent and are blocked by the SIRT1 inhibitor EX-527 (Qiu et al., 2025). Furthermore, anesthesia/surgery in aged animals activates the fructose metabolism pathway, reducing fructose-1-phosphate and increasing lactate synthesis. This lactate accumulation exacerbates cognitive impairment, whereas inhibiting this pathway lowers brain lactate levels and improves cognitive performance (Zhang L. et al., 2025). Clinically, observational findings further suggest that lactate interventions may improve cognitive outcomes in TBI patients (Bisri et al., 2016). Interventions targeting lactate availability/uptake show mechanistic support, showing that lactate supplementation or modulation can restore energy metabolism and synaptic plasticity across injury- and perioperative-related cognitive impairment models, although optimal timing and dose may be context-dependent.

**FIGURE 3:**
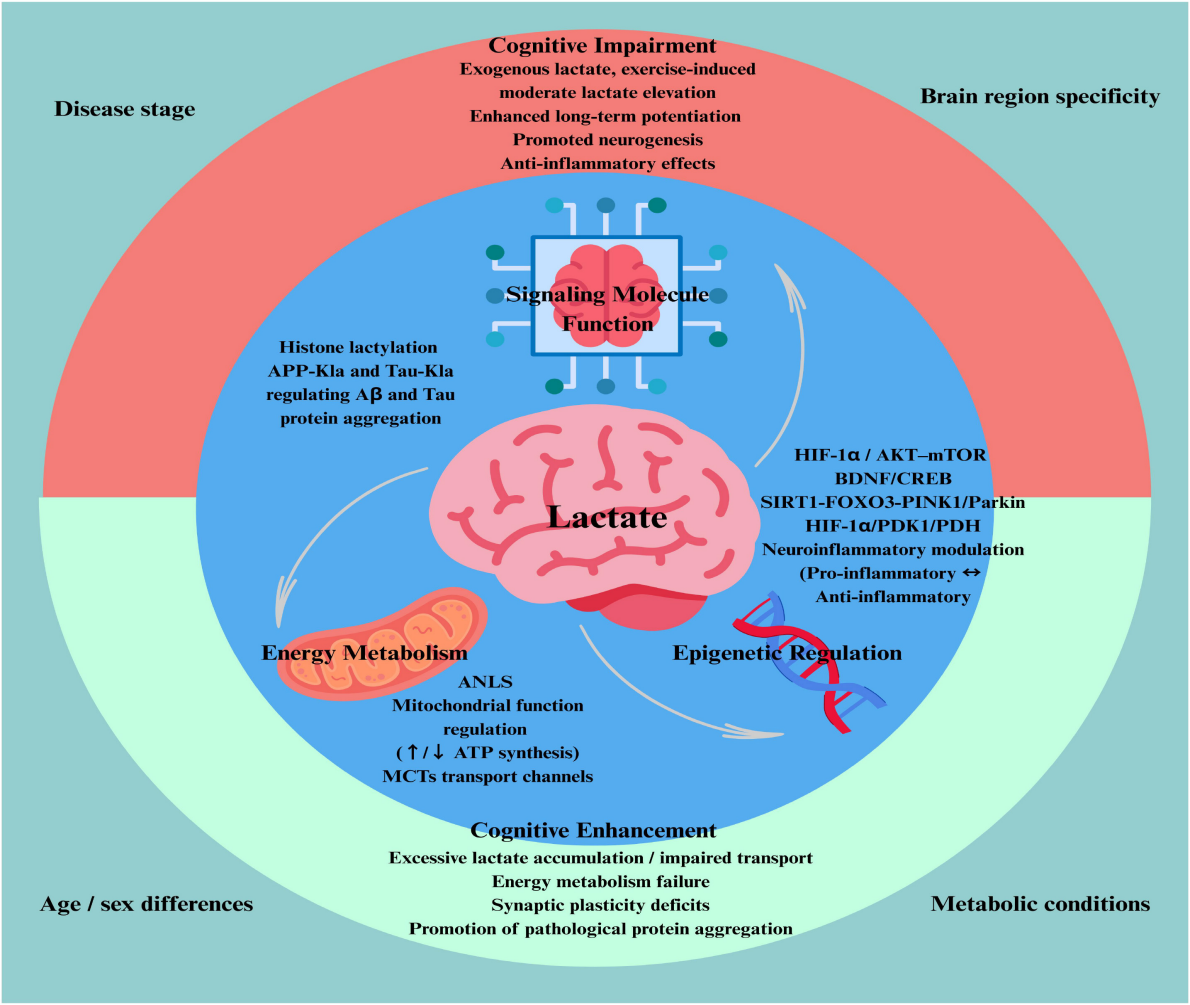
The dual role of lactate in cognitive function and its key pathways of influence. This schematic summarizes the dual roles of lactate in regulating cognitive function. Lactate acts as a key mediator of brain metabolism, serving not only as an energy substrate but also as a signaling and epigenetic regulator. Depending on concentration, duration, and physiological context, lactate can exert either beneficial or detrimental effects. Moderate increases–such as those induced by exercise–support synaptic activity, promote neuroplasticity, and confer neuroprotection. In contrast, excessive accumulation or impaired transport may result in metabolic stress, synaptic dysfunction, and cognitive decline. The net effect is shaped by factors such as disease stage, brain region specificity, age, sex, and metabolic conditions, underscoring the complexity of lactate as a double-edged regulator in cognitive health and disease.

Overall, lactate in TBI and POCD demonstrates stage-dependent, region-specific, and dose-dependent effects. In the acute phase, moderate lactate elevation or exogenous supplementation may provide metabolic support and activate neuroprotective pathways. Conversely, in certain pathological contexts (e.g., fructose metabolism dysregulation), lactate accumulation may aggravate neuronal dysfunction and cognitive impairment. These heterogeneous findings are consistent with the stage-dependent framework outlined in Figure 3 and the beneficial-versus-harmful classification summarized in Table 2.

### 3.5 Other cognitive impairments and related diseases

Lactate appears to exert age-dependent, bidirectional effects on cognition. Preclinical evidence from cellular and animal models indicates that in young rats, lactate is essential for long-term potentiation (LTP) and memory formation; inhibiting lactate production significantly impairs LTP maintenance. However, in aged animals, the role of lactate may change due to metabolic reprogramming or dysfunction in astrocyte–neuron interactions. In these cases, inhibiting lactate production can improve LTP, and lactate accumulation in the aging brain may be detrimental. Molecular mechanisms suggest that lactate influences LTP by modulating postsynaptic calcium signaling and potassium channel activity, such as the amplitude of afterpositivity (AFP), with age-dependent effects (Drulis-Fajdasz et al., 2015). Neurons rely predominantly on oxidative phosphorylation for ATP production. During normal aging, brain energy metabolism is remodeled, with PET evidence indicating a loss/alteration of aerobic glycolysis and regional reorganization of glycolytic topography, potentially compromising metabolic efficiency and neuron–glia coupling. Beyond serving as an oxidative substrate, lactate can also reflect metabolic state; under maladaptive conditions, sustained lactate dysregulation may contribute to cognitive impairment (Hall et al., 2012). The cognitive effects of lactate depend on intact transport–utilization coupling. Neuronal MCT2 is required for long-term memory formation, whereas astrocytic MCT4 contributes to information acquisition and retention. Lactate is transferred from astrocytes to neurons and plays a critical role in neuronal energy metabolism, especially in spatial learning. In experiments, lactate injection restored learning ability in MCT4-deficient mice, but MCT2-deficient mice failed to recover learning function via lactate (Netzahualcoyotzi and Pellerin, 2020). Studies also show significant sex- and age-dependent differences in neuronal lactate synthesis. Overexpression of LDHA enhances memory in young female mice but exacerbates cognitive impairment in older mice (Frame et al., 2024). Additionally, under normal cognitive conditions, lactate functions as a signaling molecule, entering neurons through MCT2, enhancing the expression of genes related to neuroplasticity (e.g., Arc, c-Fos, Zif268), and promoting neuroplasticity via the PGC1α/FNDC5/BDNF pathway to improve memory (Hwang et al., 2023), MCT2 antagonists can inhibit lactate-induced synaptic protein expression and lactylation, possibly through epigenetic regulation of synaptic protein expression, thereby enhancing synaptic plasticity proteins and neurotrophic factors (Wu et al., 2024). Furthermore, brain endothelial cells also regulate neurogenesis through lactate homeostasis (Wang et al., 2019). Both PKM2 and LDHB knockout models lead to lactate metabolic dysfunction, impairing neural stem/progenitor cell proliferation, ATP production, and cognitive behavior (He et al., 2025; Lee et al., 2025). In the anterior cingulate cortex (ACC), lactate depletion directly impairs learning, memory, and integration. Chemical genetic studies show that activating the Gi signaling pathway in ACC astrocytes reduces cAMP and lactate levels, impairing cognition. Local injection of exogenous lactate reverses these deficits and promotes mitochondrial biogenesis. Lactate enters neurons through MCT2 and enhances mitochondrial function by promoting the expression of PGC-1α, SIRT3, and ATPB, and mtDNA replication, thereby completely reversing cognitive deficits (Akter et al., 2023). Moreover, in normal brain models, lactate dynamics in the hippocampus and striatum are closely linked to the type of cognitive task. Task demands and rewards types (such as food or water) significantly influence lactate levels in these regions, and learning tasks can activate dynamic lactate changes in different brain areas, suggesting that lactate is task-specific in cognitive processing (Newman et al., 2017). Clinically, observational studies in humans further suggest that in individuals with MCI, lactate concentrations are negatively correlated with hippocampal volume, and elevated lactate levels in the posterior cingulate cortex are significantly associated with memory decline. Furthermore, lactate levels in the posterior cingulate cortex correlate negatively with brain functional connectivity, particularly with the hippocampus (Weaver et al., 2015). Large-scale population studies have confirmed that elevated plasma lactate levels are closely linked to cognitive decline, potentially serving as an intermediary biomarker between systemic inflammation and cognitive dysfunction (Pan et al., 2019). In contrast, reduced fecal lactate in dementia patients has been suggested as a metabolic reflection of altered gut microbiota and cognitive impairment (Saji et al., 2020). In patients with subarachnoid hemorrhage (aSAH), lactate levels are significantly correlated with CMD-total-tau levels; lactate plays a crucial role in the metabolic crisis of aSAH patients, with high lactate levels often linked to brain tissue hypoxia, axonal damage, and poor cognitive outcomes (Helbok et al., 2015). In schizophrenia, elevated brain lactate levels, particularly in the anterior cingulate cortex, have been negatively correlated with cognitive and functional scores. High lactate levels and altered bioenergetics likely reflect impaired aerobic metabolism and a shift to anaerobic glycolysis, possibly due to mitochondrial dysfunction (Rowland et al., 2016). In both brain tissue and patient-derived cell models, lactate levels are significantly increased, accompanied by prominent metabolic disruptions. DISC1 mutation models indicate that lactate metabolism pathways in astrocytes may play a key role in the pathophysiology of schizophrenia, with lactate level changes independent of postmortem interval, age, or brain pH, and not caused by antipsychotic medication. The lactate system may serve as a biomarker and therapeutic entry point for cognitive dysfunction in schizophrenia (Sullivan et al., 2019). Additionally, lactate levels in schizophrenia patients differ across disease stages and are closely associated with negative symptoms (Wijtenburg et al., 2021). In autism spectrum disorder (ASD) and developmental diseases, plasma lactate levels are generally elevated and negatively correlated with cognitive and adaptive abilities in children with ASD, developmental delays, and Down syndrome (Smith et al., 2023; Sotelo-Orozco et al., 2020). In these conditions, lactate accumulation may reflect mitochondrial or glial cell metabolic abnormalities, which have lasting effects on brain development.

In neurodegenerative diseases, such as Huntington’s disease and Parkinson’s disease, increased lactate synthesis combined with impaired utilization leads to lactate accumulation and cognitive deficits (Chapp et al., 2021; Chen et al., 2021; Liguori et al., 2022; Solís-Maldonado et al., 2018). In an aluminum toxicity model, aluminum exposure inhibits AMPK activity, reduces GLUT1 and GLUT3 expression, and blocks glucose uptake. This disrupts the ANLS, decreasing MCT4 and MCT2 expression, reducing lactate supply. The upregulation of HIF-1α promotes PDK1 expression, inhibits PDH activity, and impairs oxidative phosphorylation, leading to reduced ATP synthesis. Metformin can activate AMPK, upregulate GLUTs and BDNF, indirectly restore MCT2 expression, and regulate the HIF-1α/PDK1/PDH pathway, reversing cognitive impairment (Song et al., 2022). Lactate transport disorders due to AQP-4 deficiency cause hippocampal lactate accumulation, resulting in cognitive deficits (Cha et al., 2023). In α-synucleinopathies, lactate effects are modulated by catecholamine concentrations, exhibiting bidirectional effects (Shum et al., 2023). In multiple sclerosis, lactate reduction is associated with cognitive impairment (Gil-Sánchez et al., 2024). Elevated lactate in the ACC may improve decision-making by enhancing myelination and synaptic transmission (Akter et al., 2024). In insulin-resistant animals, exercise reduces lactate levels and restores cognitive pathways (Bian et al., 2024). In Mitochondrial Encephalomyopathy, Lactic Acidosis, and Stroke-like Episodes (MELAS), lactate elevation reflects metabolic crises and neurological dysfunction (Gao et al., 2024). In depression-related models, hypocretin-1 inhibits lactate and BDNF via HCRTR1, impairing neuroplasticity (Chen et al., 2024). In obstructive sleep apnea (OSA), elevated cerebrospinal fluid lactate levels are consistent with early Alzheimer’s disease-like changes. OSA disrupts brain metabolism and neurodegenerative changes through sleep fragmentation and intermittent hypoxia, but CPAP therapy can effectively reverse these effects, restoring normal biomarkers and cognitive function (Liguori et al., 2017). Interventional and translational findings further strengthen causal inference, showing that in cerebrovascular diseases, post-stroke lactate elevation is correlated with improved response inhibition (Palmer et al., 2024). In acute ischemic models, the absence of astrocytic PKM2 leads to lactate energy supply disruption, increased neuronal death, and exacerbated cognitive deficits. Exogenous lactate supplementation can reverse these effects (Kang et al., 2023). In chronic pain models, lactate depletion in the hippocampus and ACC results in cognitive impairment. Lactate’s activity-dependent release in the ACC is crucial for cognitive and decision-making behaviors. In chronic visceral pain, lactate release is suppressed, possibly contributing to decision-making dysfunction. Exogenous lactate injection improves decision-making performance in chronic pain rats and restores synaptic plasticity in the ACC, while optogenetic activation of ACC astrocytes restores lactate release and enhances decision-making abilities (Han et al., 2024; Wang et al., 2017). In neonatal hypoxia-ischemia and SNX27 mutation-induced intellectual disability and epilepsy, lactate supply or metabolic dysfunction is associated with cognitive and behavioral deficits. Lactate intervention can improve these deficits (Chu et al., 2025; Tassinari et al., 2024; Zhang et al., 2024). In MCI, lactate reduction may reflect impaired peripheral glycolytic function and mitochondrial stress, with increased ccf-mtDNA levels indicating mitochondrial damage, apoptosis, and chronic inflammation, especially in APOE-ε4 carriers (Choi et al., 2024). Integrating evidence across disorders, Figure 3 provides a unifying scaffold for interpreting when lactate is more likely adaptive versus maladaptive, while Tables 1, 2 offers an at-a-glance synthesis to support stage- and region-aware conclusions.

These observations align with the stage- and compartment-aware framework proposed above (Figure 3 and Tables 1, 2). We therefore interpret lactate as more likely adaptive when it reflects a transient/moderate increase with preserved transport–utilization capacity (e.g., intact MCT2/LDHB and efficient clearance), but more likely pathological when it indicates sustained accumulation with utilization/clearance mismatch or depletion/exhaustion of astrocyte–neuron coupling; modifiers include stage, region/compartment, age, sex, and exercise intensity/fitness (Figure 3).

## 4 Conclusion and future directions

This review evaluates the role of lactate in cognitive impairment. The evidence indicates a bidirectional impact of lactate on cognition–beneficial or detrimental–depending on lactate concentration, exposure duration, site/compartment, regional metabolic milieu, adaptive capacity, and the specific disorder/model studied. As a key mediator of astrocyte–neuron metabolic cooperation, lactate supports neuronal energy demands, and basic research further supports additional roles in receptor-mediated signaling and lactylation-related regulation that can influence neuroplasticity and synaptic function. The relationship between lactate and neuroinflammation remains model- and stage-dependent, with studies reporting both pro- and anti-inflammatory associations.

Clinically, lactate measures are mostly observational and require mechanistic and longitudinal validation. Future studies should therefore prioritize targeted, stage-aware, and translational approaches. First, modulators of lactate metabolism–targeting key enzymes (e.g., LDHA/LDHB, PKM2) or transporters (MCT1/2/4)–may help restore astrocyte–neuron coupling and improve cognitive outcomes. Second, advances in non-invasive imaging (e.g., ∧1H-MRS and lactate-based PET tracers) could enable dynamic monitoring of brain lactate metabolism for risk stratification, staging, and treatment-response tracking. Importantly, lactate is unlikely to serve as a stand-alone disease-specific biomarker; rather, disease relevance is expected to emerge from patterned lactate signatures defined by stage (prodromal/early vs. late), region/compartment (brain MRS vs. CSF vs. plasma), and transport–utilization context (e.g., MCT/LDH axis, lactate-to-pyruvate balance, inflammatory/mitochondrial markers) (Figure 3 and Tables 1, 2).

Third, the epigenetic dimension of lactate–particularly histone lactylation–warrants deeper investigation to clarify its roles in synaptic plasticity, neuroinflammation, and cognitive regulation. Moreover, rigorously designed clinical trials are needed to establish causal links between lactate manipulation (supplementation, exercise, dietary modulation) and cognitive outcomes. Lactate-based interventions should not be presumed universally beneficial: they may be adaptive in depletion phenotypes but detrimental in accumulation/mismatch phenotypes, making dose, timing, and patient stratification essential. Practical guidance for stage- and context-dependent lactate modulation is summarized in Table 2 and aligns with the framework in Figure 3.

Collectively, prospective, stage-stratified and region-resolved studies are required to validate actionable lactate signatures, define safe therapeutic windows, and enable clinically meaningful translation (Figure 3 and Tables 1, 2). In summary, lactate should be interpreted as a stage- and region-dependent mediator of cognition (Figures 1, 3 and Tables 1, 2).
